# Brian Benedetti-Williams, 1956–2016

**DOI:** 10.1038/cddiscovery.2016.91

**Published:** 2017-01-23

**Authors:** M Benedetti-Johnson


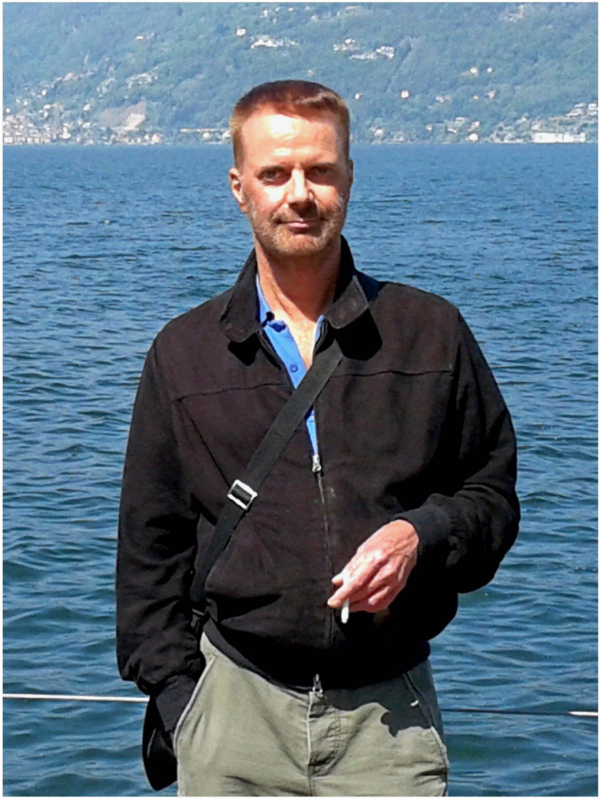

*Animula vagula, blandula,*

*Hospes comesque corpis,*

*Quae nunc abibis in loca*

*Pallidula, rigida, nudula,*

*Nec, ut soles, dabis iocos*

*P. Aelius Hadrianus, Imp.*

[Piccola anima smarrita e soave, compagna ed ospite del corpo, ora ti appresti a scendere in luoghi incolori, ardui e spogli, ove non avrai più gli svaghi consueti. Un istante ancora, guardiamo insieme le rive familiari, le cose che certamente non vedremo mai più ….cerchiamo d’entrare nella morte a occhi aperti….]

[Little soul, you charming little wanderer, my body's guest and partner, where are you off to now? Somewhere without colour, savage and bare; you'll crack no more of your jokes once you're there].

‘*I am not afraid of death—I am privileged to have been able to work for so long,’ said Levi-Montalcini. ‘If I die tomorrow or in a year, it is the same—it is the message you leave behind you that counts, and the young scientists who carry on your work*.’

On April 2016, Brian passed away in London aged 59 years old. Brian was a dear friend and an inspiration to his generation not only because of his professional achievements but also for his warm personality, exemplary hard-playing life and unbounded enthusiasm.

A polymath, post-enlightenment ethos flowed to all his friends, creating an ambience where intellectual excellence was highly appreciated and avidly pursued. Brian’s mother inspired him with Italian culture, a lifelong passion that he was able to explore in depth during his long stay in Venice. He was indeed a keen ‘child of the Renaissance’ immersed in literature and the humanities, displaying extraordinary literary talents, whereas his attitude to life was always at the extreme. Work hard and play hard was his rule, and he really did that! He experienced every kind of pleasure and relationships regardless of any conventional ethical, medical or legal formality. His whole approach to life was to plunge headlong into new experiences.

After completing grammar school, the young Brian felt unable to adjust to the conventional suburban Romford role (expected by his father) and embarked, instead, on a career in psychology and medical research. In this, he achieved an excellent standard, the science complementing his literature interests, and earning him the affection and respect of friends and colleagues.

In the seventies, Brian established himself in London, where I had the pleasure of meeting him. Here, indeed, I had the great honour to have a close interaction with Brian, admiring his curiosity, his desire to keep abreast of knowledge, and ability to inspire enthusiasm and loyalty among all his many friends, characteristics that persisted to his very last day. In simple words, Brian was a model to his friends and a model for life. Always a big laugh with his venetian jokes:

*Ghe xe na cupia de laorenti che i no ghe ‘l fa a meter a ‘l mondo un fio. El marido sa che ea mujer ga un caxin de problemi—prexempio ea xe purasé sorda confà na cioca. Eaora el deçìde che ea mujer cogna fazer na visita specialistica co’ el jinecologo. Adonca, iori i ariva a ‘l studio de ‘l medego e ea mujer intra indrento. (Par zonta ea muga el italian.) El medego (che xe italian) ghè fa un controio total e può se pronunza: «Lei ha un polipo che cresce—un adenoma. Se Lei ce la facesse a fare un bambino sarebbe un miracolo!». Eaora, ea mujer torna desfita da ‘l marido—che el sta cogna anxioso in ea sala de ateza e ghè dimanda dibot «Che te ga dito el medego?» Ea ghè ribata «Mi ga dito che mi stago co’ na polpeta de pese in ea mona e se fasso un toseto saria un macareio!*»

Brian was ‘one of a kind’ in the deepest and broadest sense of these words, very close to me in person. Hard to imagine that this hard-living, hard-playing and exaggerated man—the ultimate in fine taste—carved his way with an incredible spirit, friendship and humanity. He will always live in our hearts. We will miss him sorely. My thoughts and deepest sympathy are with you Brian, my greatest love of all, at this sad time.

